# Bleeding Complications from Chest Tube Treatment in Patients on Extracorporeal Membrane Oxygenation Support

**DOI:** 10.1093/icvts/ivaf271

**Published:** 2025-11-22

**Authors:** Axel Dimberg, Magnus Dalén, Lars Mikael Broman, Ulrik Sartipy, Magnus Larsson

**Affiliations:** Department of Cardiothoracic Surgery, Karolinska University Hospital, 141 86, Stockholm, Sweden; Department of Molecular Medicine and Surgery, Karolinska Institutet, 171 64, Stockholm, Sweden; Department of Cardiothoracic Surgery, Karolinska University Hospital, 141 86, Stockholm, Sweden; Department of Molecular Medicine and Surgery, Karolinska Institutet, 171 64, Stockholm, Sweden; ECMO Centre Karolinska, Department of Pediatric Perioperative Medicine and Intensive Care, Karolinska University Hospital, 141 86, Stockholm, Sweden; Department of Physiology and Pharmacology, Karolinska Institutet, 171 64, Stockholm, Sweden; Department of Cardiothoracic Surgery, Karolinska University Hospital, 141 86, Stockholm, Sweden; Department of Molecular Medicine and Surgery, Karolinska Institutet, 171 64, Stockholm, Sweden; ECMO Centre Karolinska, Department of Pediatric Perioperative Medicine and Intensive Care, Karolinska University Hospital, 141 86, Stockholm, Sweden

**Keywords:** ECMO, chest tube, pneumothorax, pleural fluid, bleeding complications

## Abstract

**Objectives:**

Bleeding is a common complication of extracorporeal membrane oxygenation (ECMO) support. While chest tube treatment is infrequently linked to bleeding in non-ECMO patients, several reports suggest a markedly increased risk during ECMO support, sometimes requiring surgical intervention. The true incidence, contributing factors, and effect on outcome from chest tube related bleeding in ECMO patients is unknown.

**Methods:**

This was a single-centre observational study conducted between 2010 and 2024, including both paediatric and adult patients treated with chest tube for pneumothorax or non-haemorrhagic pleural fluid during their ECMO treatment. Major bleeding was defined by chest tube output amount adjusted to patient age, need for multiple red blood cell transfusions, or need for surgical intervention.

**Results:**

Of the 1158 screened ECMO patients, 168 (14.5%) required chest drainage for pleural effusions or pneumothorax during the study period, and a total of 279 chest tubes were analysed. Major bleeding occurred in 21 patients (12.5%) and from 23 chest tubes (8.1%). Fourteen patients required thoracotomy. Bleeding was more common with tubes placed during ECMO support (11.3%) compared to pre-ECMO (4.7%, *P* = .036). Affected patients had longer ECMO durations (median 42 vs 17 days, *P* = .003) and lower hospital survival (47.6% vs 71.4%, *P* = .043). No associations were observed between bleeding and tube size, laterality, type, insertion technique, or ECMO mode. The chest wall was the most commonly identified bleeding location.

**Conclusions:**

There was a markedly increased risk of major bleeding from chest tubes during ECMO, particularly when inserted after cannulation. Patients who experienced bleeding complications had longer hospital stays and lower survival.

## Graphical abstract

**Figure:**
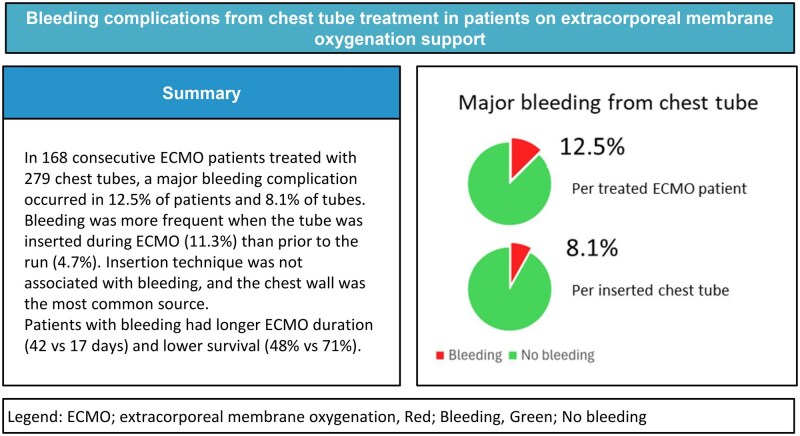

## INTRODUCTION

Up to half of patients on extracorporeal membrane oxygenation (ECMO) experience bleeding complications.^1–3^ Pleural collections of air or fluid are common during ECMO treatment and often lead to intervention with chest tube drainage.^4^ Chest tube thoracostomy is rarely associated with major bleeding in non-ECMO populations.^5^^,^^6^ During ECMO, however, several studies mention severe bleeding complications linked to chest tube treatment, sometimes necessitating thoracotomy for haemostasis and evacuation.^4^^,^^7–9^ Few studies specifically investigate chest tube treatment on ECMO. One study reviewing chest tube treatment in paediatric ECMO patients reported major or fatal iatrogenic pleural bleeding in 6 of 27 patients receiving drainages while on ECMO.^10^ Another group examined all ECMO patients undergoing thoracotomy and found excessive bleeding post chest drainage insertion preceded surgery in 11 of 19 identified cases.^11^

Although severe chest tube-related bleeding during ECMO is frequently reported, the exact incidence, risk factors, and impact remain unclear.

This study aimed to determine the incidence, severity, and consequences of major chest drainage-related bleedings in ECMO patients. Secondary objectives included examining associations between major bleeding events, patient characteristics, and individual chest tube parameters including number of chest tubes, indication, size, and insertion technique.

## METHODS

### Ethical statement

This study was approved by the Swedish Ethical Review Authority (registration numbers 2022-01073-01 and 2024-06366-02). The board approved that the requirement for informed consent was waived due to the anonymity and retrospective design of the study.

### Patient selection

This was an observational single-centre study evaluating incidence and characteristics of major bleeding events caused by chest tube treatment during ECMO. The study included patients treated between 2010 and 2024 at our centre which is an international referral ECMO unit also providing mobile ECMO services for primary, secondary, and tertiary ECMO transports of all age groups. The centre treats all types of diagnoses but operates independently of the cardiothoracic intensive care unit and therefore generally does not treat post-cardiotomy patients.

Patients were included if they had a chest drainage in place during ECMO support, regardless of if it was inserted before or during ECMO. Drainages placed for suspected pleural fluid, pus, or pneumothorax were included. Drainages for pre-existing haemothorax were excluded. All age groups, ECMO modes (veno-arterial, veno-venous, conversions or hybrid), and underlying diagnoses were eligible for inclusion except congenital diaphragmatic hernia due to frequent thoracic surgery and drainage requirements. Patients undergoing thoracotomy or sternotomy for other indication than chest tube-related haemothorax, eg lung biopsy or cannulation complications, were excluded.

Cases with drainages were identified from the local ECMO quality database and electronic medical records. Records were systematically screened using Swedish Classification of Healthcare Surgical Procedures codes for thoracic drainages (GAA10, GGA96, TGA30, TGA35), and free text search terms. Collected data included indication (pneumothorax or fluid, including pus), drainage type, laterality, size, insertion technique, and description of bleeding complications. Missing information on drainage type, size, laterality, or indication was supplemented through review of chest x-ray examinations. Two physicians experienced in ECMO treatment extracted the data and evaluated radiological findings.

Chest tubes placed during ECMO were inserted by experienced intensive care physicians and surgeons at a specialized ECMO centre, while chest tubes placed before the start of ECMO were also inserted at other departments or hospitals. Patients on ECMO were generally treated with a standard anticoagulation protocol using unfractionated heparin infusion, targeting an activated partial thromboplastin time (aPTT) of 1.5 to 2.5 times above upper reference level, typically 45-80 seconds, although anticoagulation treatment was highly individualized depending on coagulopathy, diagnosis, ECMO mode, and bleeding status.

### Outcomes

The primary outcome was the occurrence of a major bleeding event caused by chest tube treatment. Baseline patient characteristics and treatment outcomes, including survival to decannulation and hospital discharge, were compared between chest tube treated patients with and without bleeding. In a separate analysis, chest tube characteristics were compared between individual drainages causing or not causing bleeding, respectively.

### Definition of bleeding

Major bleeding was defined as a chest tube haemorrhagic output over 24 hours exceeding 800 mL in adults, 20 mL/kg in paediatric, and 30 mL/kg in neonate patient, a bleeding necessitating more than 2 transfusions of packed red blood cells (PRBC) due to circulatory instability, radiographic evidence of massive haemothorax, or bleeding requiring surgical intervention. To avoid misclassifying spontaneous haemorrhages as iatrogenic events, bleeding was attributed as related to the drainage when the initial chest tube output consisted of air or non-haemorrhagic pleural fluid, and bleeding started within 24 hours after drainage insertion. Events beyond 24 hours were included only if active drain-related bleeding was confirmed by direct visualization (externally via the drainage insertion site or intraoperatively during thoracotomy), or contrast-enhanced computed tomography demonstrating extravasation adjacent to the drainage insertion involving the chest wall or parenchymal organ. Patients not fulfilling these criteria were assigned to the non-bleeding group. In uncertain cases, a conservative approach was adopted to exclude potential cases of spontaneous bleeding.

### Missing data

There was missing data of size or type concerning 5 chest tubes (1.8%) and indication was missing in 1 case (0.4%).

### Statistical analysis

Data are presented as mean (SD), median [IQR], or *n* (%). For age and weight in the total cohort, both mean (SD) and median [IQR] are reported, whereas subgroup values are shown as mean (SD). Normality was assessed with Shapiro-Wilk tests and inspection of histograms. Bivariate logistic regression estimated odds ratios (ORs) with 95% CIs to quantify the unadjusted association between predictors and major bleeding. Continuous variables were compared using the Mann-Whitney *U* test, and categorical variables using Fisher’s exact test. A *P*-value < .05 was considered statistically significant.0 (StataCorp LLC, College Station, TX, USA).

## RESULTS

**Figure 1.**
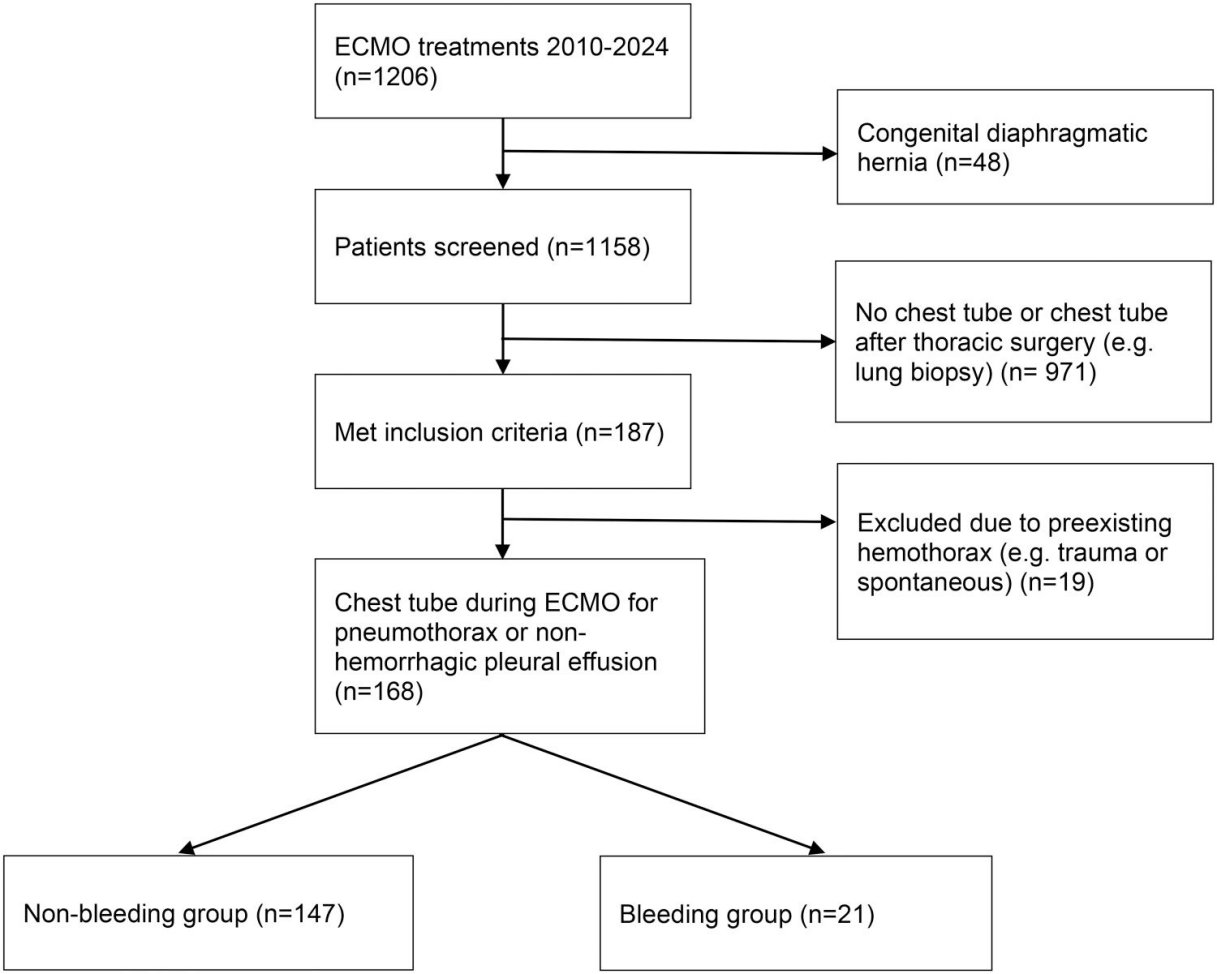
Flow Chart (ECMO—Extracorporeal Membrane Oxygenation)

The study population consisted of 168 patients who had a chest tube in place during ECMO treatment due to suspected pneumothorax or non-haemorrhagic pleural effusion, corresponding to 14.5% of the 1158 screened patients (**Figure 1**). A total of 279 drainages were placed, and the mean number of drainages per patient was 1.7. Multiple chest tubes were common: 55 patients (32.7%) received 2, and 23 (13.7%) received 3 or more. In 67 patients, drainages were only placed prior to ECMO initiation (**Table 1**). The group composition was 43% female with a mean age of 26.6 (SD 24.6) years. Neonates (0-28 days) comprised 23%, paediatric patients 24%, and adults (≥18 years) 53%.

**Figure 2.**
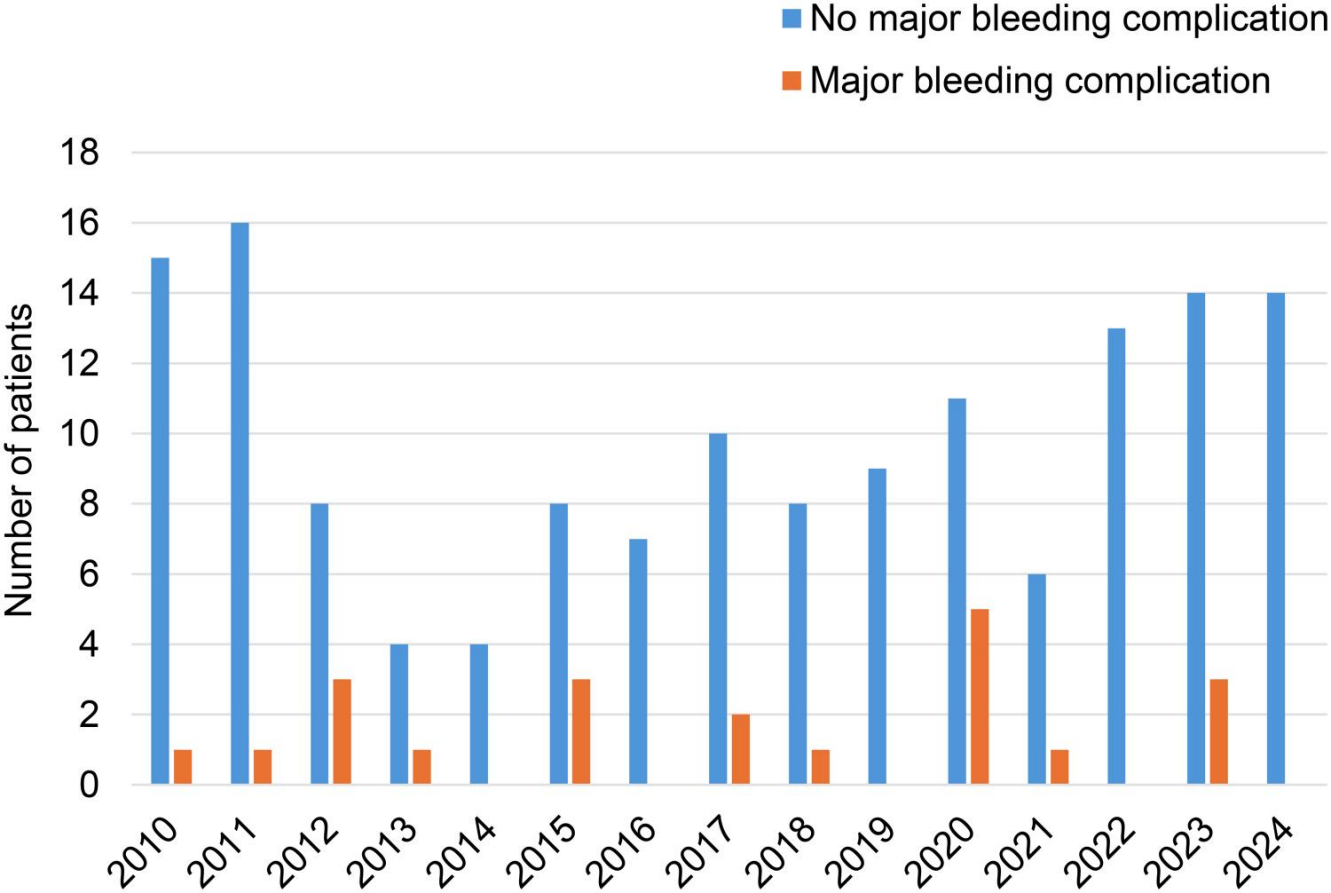
Annual Number of Patients Treated With Chest Tube for Pneumothorax or Pleural Fluid While on Extracorporeal Membrane Oxygenation per Year

**Table 1.** Baseline Characteristics in Patients Treated With Chest Tube During Extracorporeal Membrane Oxygenation Categorized on Bleeding Complications

Variable	Total population	Non-bleeding	Major bleeding	*P*-value
Patients (*n*)	168	147	21	
Age, years, mean (SD); median [IQR]	26.6 (24.6)	25.0 (24.3)	37.8 (24.3)	.036
	22 [1-52]	9 [0-52]	42 [17-55]	
Neonatal, days	2.7 (4.6)	2.6 (4.7)	3.4 (3.8)	.57
Paediatric, years	6.4 (5.2)	5.9 (4.8)	12.8 (6.8)	.0498
Adult, years	47.3 (14.3)	46.7 (14.2)	50.4 (14.9)	.50
Age group, *n* (%)				.238
Neonatal group	39 (23.2%)	36 (24.5%)	3 (14.3%)	
Paediatric group	40 (23.8%)	37 (25.2%)	3 (14.3%)	
Adult group	89 (53.0%)	74 (50.3%)	15 (71.4%)	
Female sex, *n* (%)	72 (42.9%)	67 (45.6%)	5 (23.8%)	.064
Weight, kg, mean (SD); median [IQR]	50.8 (39.6)	48 (40.1)	65.5 (33.9)	.08
	57 [7-82]	51 [5-82]	72 [58-82]	
Neonatal	3.7(0.8)	3.6 (0.8)	4.5 (0.4)	.047
Paediatric	24.7 (19.5)	23.0 (18.3)	45.7 (25.5)	.09
Adult	83 (2.1)	83.5 (21.8)	81.7 (19.7)	.62
Diagnosis, *n* (%)				.038
Cardiac^a^	23 (13.7%)	23 (15.6%)	0 (0%)	
COVID-19	14 (8.3%)	9 (6.1%)	5 (23.8%)	
Pneumonia/ARDS	65 (38.7%)	55 (37.4%)	10 (47.6%)	
Sepsis	32 (19%)	28 (19.1%)	4 (19.1%)	
Meconium aspiration	21 (12.5%)	20 (13.6%)	1 (4.8%)	
Other^b^	13 (7.7%)	12 (8.2%)	1 (4.8%)	
Year of treatment, *n* (%)				.33
2010-2014	53 (31.6%)	47 (32.0%)	6 (28.6%)	
2015-2020	64 (38.1%)	53 (36.0%)	11 (52.4%)	
2021-2024	51 (30.4%)	47 (32.0%)	4 (19.1%)	

a Cardiac included cardiogenic shock and extracorporeal cardiopulmonary resuscitation.

b Other included acute myeloid leukaemia, drowning, oesophageal perforation, foreign body in trachea, intoxication, lung fibrosis, trauma, and Wegener’s granulomatosis.

Abbreviations: ARDS, acute respiratory distress syndrome; IQR, interquartile range; SD, standard deviation.

Major bleeding directly caused by a chest tube occurred in 21 of 168 patients (12.5%) (**Figure 2**). Fourteen of these 21 patients (66.7%) underwent at least one thoracotomy due to bleeding, with a total of 39 thoracotomies performed. Two patients experienced separate unrelated bleeding episodes, resulting in a total of 23 events in 21 patients. Each bleeding event is described in detail in **Table S1**. The most common bleeding source was the chest wall at the site of the drainage passage, identified in 14 of 23 events through extravasation on CT or direct visualization during thoracotomy or externally. In 4 patients, the chest tube-related bleeding was considered as the main cause of death (**Tables S2 and S3**).

Patients with bleeding had longer ECMO duration (42 vs 17 days, *P* = .003) and lower survival to hospital discharge (47.6% vs 71.4%, *P* = .043) compared to those without bleeding (**Table 2**). The patients experiencing bleeding were also older (37.8 vs 25.0 years, *P* = .036), and had more drainages placed (2.6 vs 1.6). Diagnosis category differed significantly between groups (*P* = .038). Notably, pneumonia or acute respiratory distress syndrome and COVID-19 cases were more common among bleeding cases, while no bleeding events occurred in patients within the cardiac diagnosis group. Sex, weight, and treatment year did not differ significantly between groups.

**Table 2.** Treatment Outcome and Complications

Variable	Total population	Non-bleeding	Major bleeding	*P*
Patients (*n*)	168	147	21	
Time on ECMO, days (SD)	20.3 (28.4)	17.1 (18.0)	42.3 (61.5)	.003
VV ECMO only, *n* (%)	57 (33.9%)	48 (32.7%)	9 (42.9%)	.46
VA ECMO or combinations, *n* (%)	111 (66.1%)	99 (67.3%)	12 (57.1%)	.46
Survival to weaning, *n* (%)	120 (71.4%)	109 (74.1%)	11 (52.4%)	.067
Survival to hospital discharge, *n* (%)	115 (68.5%)	105 (71.4%)	10 (47.6%)	.043
ECMO transport type, *n* (%)				.25
No transport	32 (19.0%)	28 (19.0%)	4 (19.0%)	
Air	81 (48.2%)	74 (50.3%)	7 (33.3%)	
Ground	55 (32.7%)	45 (30.6%)	10 (47.6%)	
Number of thoracic drains, mean (SD)	1.7 (0.9)	1.6 (0.8)	2.6 (1.2)	<.001
Drains before start of ECMO	0.8 (1.0)	0.8 (0.9)	0.9 (1.2)	.70
Drains on ECMO	0.9 (1.0)	0.8 (0.9)	1.7 (1.3)	.001
Complications from drain, *n* (%)				
Organ perforation (spleen,^a^ lung,^a^ heart, liver)	4 (2.4%)	2 (1.4%)	2 (9.5%)	
Thoracotomy due to bleeding	-	-	14 (66.7%)	

a Did not cause major bleeding.

Abbreviations: ECMO, extracorporeal membrane oxygenation; SD, standard deviation; VA, veno-arterial; VV, veno-venous.

Major bleedings occurred in 23 of the 279 individual chest drainages (8.1%). Bleeding was less frequent from chest tubes placed before ECMO initiation: 6 of 128 (4.7%) drainages inserted pre-ECMO caused bleeding later during ECMO support compared to 17 of 151 (11.3%) of the chest tubes placed during ECMO (*P* = .036). No significant associations were found between bleeding and drainage characteristics, including laterality, type, insertion technique, indication (pneumothorax versus pleural fluid), time from or ECMO mode at implantation (**Table 3**).

**Table 3.** Individual Chest Tubes Placed on ECMO, Categorized From Bleeding Complications (2 Patients Experienced 2 Complications)

Variable	Total chest tubes	No bleeding	Bleeding	*P*	Missing, *n*
Chest tubes (*n*)	279	256	23		
Drain insertion timing, *n* (%)				.036	0
Before ECMO, *n* (%)	128 (45.9%)	122 (47.7%)	6 (26.1%)		
On ECMO, *n* (%)	151 (54.1%)	134 (52.3%)	17 (73.9%)		
Mode at time of drain insertion, *n* (%)				.09	0
Pre ECMO	128 (45.9%)	122 (47.7%)	6 (26.1%)		
VV ECMO	54 (19.4%)	47 (18.4%)	7 (30.4%)		
VA ECMO	97 (34.8%)	87 (34.0%)	10 (43.5%)		
Time on ECMO at drain insertion, days (SD)					0
Drains placed before start of ECMO	-1.46 (3.1)	-1.52 (3.2)	-0.3 (0.52)	.092	
Drains placed on ECMO	13.5 (14.1)	13.3 (14.2)	15.0 (13.0)	.39	
Laterality, *n* (%)				.66	0
Right side	172 (61.6%)	159 (62.1%)	13 (56.5%)		
Left side	107 (38.4%)	97 (37.9%)	10 (43.5%)		
Indication for drain, *n* (%)				1.00	1 (0.4%)
Pneumothorax	136 (48.7%)	125 (48.8%)	11 (47.8%)		
Pleural fluid	129 (46.2%)	118 (46.1%)	11 (47.8%)		
Previous drain malfunction or incorrect diagnosis^a^	13 (4.7%)	12 (4.7%)	1 (4.3%)		
Missing/unknown	1 (0.4%)	1 (0.4%)	0 (%)		
Type of drain, *n* (%)				.85	
Standard chest tube (open)	109 (39.1%)	100 (39.1%)	9 (39.1%)		5 (1.8%)
Seldinger chest tube (guide wire)	90 (32.3%)	81 (31.6%)	9 (39.1%)		
Pigtail drainage/CVC/PVC	75 (26.9%)	70 (27.3%)	5 (21.7%)		
Missing/unknown	5 (1.8%)	5 (2.0%)	0 (0%)		
Drain size, Fr (SD)	11.7 (6,4)	11.6 (6.2)	13.1 (7.5)	.44	4 (1.4%)
Standard chest tube	15.5 (6.4)	15.2 (6.4)	19.3 (5.5)	.048	
Seldinger chest tube	11.8 (4.6)	11.8 (4.5)	11.5 (5.5)	.62	
Pigtail/CVC/PVC	6.0 (3.2)	6.1 (3.2)	4.8 (3.3)	.26	
Drain size, Fr (SD)	11.7 (6,4)	11.6(6.2)	13.1 (7.5)	.44	4 (1.4%)
Neonatal	9 (2.8)	9.1(2.7)	7.7 (4.5)	.58	
Paediatric	9.6 (4.7)	9.6 (4.7)	10.7 (4.6)	.79	
Adult	13.9 (7.3)	13.8 (7.3)	14.5 (8.0)	.73	

a Incorrect diagnosis, eg misinterpreted pneumothorax on x-ray.

Abbreviations: CVC, central venous catheter; ECMO, extracorporeal membrane oxygenation; Fr, French (1 Fr = 1/3 mm outer diameter); PVC, peripheral venous catheter; SD, standard deviation; VA, veno-arterial; VV, veno-venous.

## DISCUSSION

The main finding of this study was the high incidence of drainage-related major bleeding events, often leading to thoracotomy. Major bleedings occurred in 12.5% of treated patients and from 8.1% of individual chest tubes. Bleeding was significantly more common from drainages inserted during ECMO (11.3%) compared to those placed before initiation (4.7%). Patients experiencing bleeding had longer ECMO duration, lower survival rates, and were more commonly treated for respiratory infections, such as pneumonia, acute respiratory distress syndrome, sepsis, or COVID-19. However, no associations were found between bleeding events and chest tube characteristics such as size, insertion technique, indication, or ECMO modality.

To the best of our knowledge, no previous publication has specifically investigated chest tube-related bleeding complications in ECMO patients across a large, mixed-age cohort. We believe this study fills an important knowledge gap. It is also the first study to describe each placed drainage and its characteristics. This is important, as many patients receive more than 1 chest tube, and insertion techniques vary.

Our results concur with those of Jackson et al who also identified a chain of events from patients receiving a chest tube leading to repeated thoracotomy and in some cases also mortality.^10^ In our study, 6 out of 79 paediatric and neonatal patients experienced bleedings which was less than a third of the findings by Jackson et al (6 out of 25). It is possible that a cluster of bleeding events in a single centre prompted analysis and publication, introducing reporting bias from both Jackson’s and this study.

Joshi et al^11^ reported 11 thoracotomies due “excessive bleeding post chest drain insertion” among 569 adult ECMO patients, and we identified 14 among 1158. These figures are not directly comparable due to the low event rate and may instead reflect institutional thresholds for surgery rather than true bleeding incidence. Many strategies can be considered when managing a patient with haemothorax on ECMO. With ongoing bleeding, interventional radiology with stents or coiling can be considered. If the bleeding stops after coagulation optimization, the remaining haemothorax can be treated with surgery, additional chest tube, irrigation and thrombolysis, conservative management, or even delayed intervention after ECMO. If overall prognosis poor, surgical intervention could be withheld.

Chest tube usage during ECMO was 14.5% of patients, which was much more common than the 3.6% reported by Tashiro et al in a paediatric cohort.^12^ Tashiro’s lower incidence likely reflects their exclusion of pre-ECMO and insertions after 8 days. In our cohort, 46% of chest tubes were inserted before ECMO implantation, and those placed during ECMO took place at a mean of 13.5 days into ECMO support. As such, majority of our cases should have been excluded by those criteria. Interestingly, Tashiro found mortality not to be higher among patients treated with chest tubes after propensity score matching.

It is difficult to draw conclusions about bleeding risk factors and outcomes in a non-randomized study with only 23 events, particularly given the heterogeneity of ECMO patients and the varying indications for chest tube placement. However, the chest tube insertion procedure itself is a relatively uniform procedure. As expected, given the coagulation challenges during ECMO, bleeding was more common from chest tubes inserted during ECMO than from those already in place at the time of cannulation. Importantly, tubes placed before ECMO did not eliminate bleeding risk. Therefore, the potential benefits from a chest tube in a patient who is close to ECMO treatment should also be contrasted to the risk of later major bleeding, and if considered, placed as atraumatic as possible.

In non-ECMO populations, severe chest tube bleeding is rare: one group reported no bleeding incidents from 373 tube thoracostomies in emergency care,^5^ and another study only found one case due to lung laceration leading to thoracotomy out of 599 chest tubes.^13^ Bleeding incidences from chest tubes are uncommon also in patients with elevated international normalized ratio (INR).^6^ Furthermore, a large meta-analysis of patients with uncorrected coagulopathy due to disease or antithrombotic drugs reported a bleeding rate of 0.71% from chest tube thoracostomy and thoracentesis.^14^

Haemorrhagic death has been reported in 3.9% of non-cardiac paediatric ECMO patients in the Extracorporeal Life Support Organization (ELSO) registry from 2002 to 2013.^15^ Data from our own department showed that 21% of adults experienced intracranial haemorrhage with >80% 6-month mortality.^16^ Spontaneous extracranial bleeding was found to be a risk factor. In addition, higher aPTT levels have been observed in bleeding patients compared to those without bleeding, suggesting that coagulopathy plays a significant role^3^; however, management of heparin anticoagulation may be an important confounder.

The need for anticoagulation during ECMO to prevent circuit-induced thrombosis inherently increases bleeding risk.^17^ However, the ECMO patients are coagulopathic for several reasons. The ECMO circuit itself affects haemostasis, potentially causing simultaneous thrombosis and bleeding events through platelet activation, coagulation factor consumption, or anticoagulant therapy.^18–20^

Initiation of unfractionated heparin anticoagulation reduces platelet count. Platelets and von Willebrand factor (vWF) are crucial for thrombus formation. Platelets are activated by adhesion molecules such as collagen, fibrinogen, vWF, and platelet agonists (adenosine diphosphate, thrombin, thromboxane A_2_). vWF functions primarily as a mediator for platelet adhesion and as a carrier protein for factor VIII. Under conditions of high shear stress, vWF unfolds, exposing multiple platelet-binding sites and facilitating thrombus formation.^21^ However, further exposure to shear stress may lead to loss of vWF capacity to bind platelets and increased risk of bleeding. ECMO support thus contributes to both acquired von Willebrand syndrome and impaired platelet function.^22^^,^^23^

Although prior studies report more bleedings in VA than VV ECMO,^1^^,^^24^ we found no such difference for chest tube-related bleedings. This suggests other factors, such as diagnose leading to ECMO need, to play a larger role than ECMO-mode *per se* for risk of chest tube-related bleedings.

The patients in this study, bleeding or not, had notably longer ECMO courses (mean 17.1 days) than usually reported. One major cause for this was likely the study period spanning over the COVID-19 pandemic during which our local database showed mean days on ECMO 22 (range 5-132 days). Adding to this was likely also the need for a chest tube marking more severe disease including pneumonia-related pleural effusion, or ventilator associated pneumothorax. The need for drainage often developed after about 2 weeks of ECMO support, with additional problems to solve and, thus, prolonging time for healing, and subsequent weaning of ECMO.

We have recently adopted the following routines during chest tube insertion for patients on ECMO. Heparin infusion is, if possible, paused for up to 6 hours before the procedure. Computerized tomography of the chest is mandatory in any non-acute case for correct diagnosis and precise localization of pleural collection. Ultrasound-guidance is used for choosing intercostal space and location. The ventilator is paused at time of puncture until guidewire in place. First puncture is performed with a 5-French (Fr) diameter Seldinger micro-puncture kit, then switch to standard guidewire for placement of a drainage using the Seldinger technique. It is preferred to use small diameter drainages (8-12 Fr).

To avoid thoracotomy for haemothorax in the acute phase of ongoing chest tube related bleeding, first attempt is angiography with coil embolization. In our experience, open chest surgery for haematoma evacuation frequently triggers a cascade of repeated thoracotomies due to bleeding aggravation by the procedure itself. When active bleeding has stopped, and a persistent haemothorax cannot be drained via additional chest tubes, we consider using continuous irrigation and intrapleural fibrinolysis with recombinant tissue plasminogen activator. Intrapleural fibrinolysis during ECMO has been described by others as an appealing alternative to thoracotomy^9^ and used successfully by us in several cases including in patients in this study.

Most importantly, we evaluate the need for each chest tube in the treatment team before insertion. Many cases can be treated conservatively, and we try to use chest tubes only in cases where it is imperative, such as when a pleural effusion create pumping problems, or believed to be major cause for weaning problems, eg lung compression or mediastinal shift.

### Limitations

This study has its inherent limitations due to its observational design, modest event count, and unrecorded confounders (anticoagulant treatment, coagulation biochemistry, ventilator settings, level of mobilization, etc). Data on other complications (pneumothorax, infection, dislocation, malfunction) were not recorded, as they were not within the scope of this study. Unfortunately, information regarding ultrasound guidance during the insertion procedure was not routinely documented in the medical records and was therefore excluded from this study. Other important limitations are the heterogeneity of the population and the long study period. Both adult and paediatric patients were included, supported with either venoarterial or venovenous ECMO and for a wide range of diagnoses. These factors may have influenced both the incidence and outcomes of bleeding complications and limit the generalisability of the results to specific subgroups of ECMO patients.

## CONCLUSIONS

Chest tube treatment during ECMO carries a substantial risk of major bleeding. While drainages placed both before and after ECMO initiation can result in bleeding, the risk is higher when insertion occurs during ECMO. In this study, each drainage placed during ECMO was associated with an 11.3% risk of major bleeding, and major bleeding was associated with severe complications. These findings support a conservative approach to chest tube insertion in patients in whom ECMO treatment is ongoing or being considered.

## Supplementary Material

ivaf271_Supplementary_Data

## Data Availability

The data underlying this article are available from the corresponding author upon request. Data are not publicly available due to ethical restrictions.
