# Supplementary material for: Bleeding Complications from Chest Tube Treatment in Patients on Extracorporeal Membrane Oxygenation Support
Source: Interdiscip Cardiovasc Thorac Surg. 2025 Nov 22;40(11):ivaf271. doi: 10.1093/icvts/ivaf271 (PMC12653765; doi:10.1093/icvts/ivaf271)
Supplement: ivaf271_Supplementary_Data [file ivaf271_supplementary_data.zip › Supplementary Table 1.docx]

| **Supplementary table 1.** Description of major iatrogenic bleeding events from chest tube treatment | | | | | | |
| --- | --- | --- | --- | --- | --- | --- |
| Patient | Age | Diagnosis | Description | Source | Drain type | Survived |
| 1 | Pediatric | Sepsis | Septic patient with thrombocytopenia, bilateral pneumothorax treated a few hours pre-ECMO, moderate left-sided bleeding worsened after ECMO initiation with transfusion need 4 units/h, thoracotomy at 12 h evacuated 5 L hematoma. | Chest wall  (seen at thoracotomy) | 8 Fr standard chest tube | Yes |
| 2 | Adult | Pneumonia/ARDS | Treated for tension pneumothorax pre-ECMO, bleeding began after 4 days with volume up to 4 L/day, thoracotomy. | Chest wall (seen at thoracotomy) | 24 Fr standard chest tube | No |
| 3 | Adult | Pneumonia/ARDS | Clear pleural fluid drained via CVC, shortly after massive hemothorax with mediastinal shift; new chest tube drained 2.4 L. Three thoracotomies due to ongoing bleeding, source could not be identified. | Unknown | 3 Fr CVC needle | No |
| 4 | Adult | Pneumonia/ARDS | Treated for tension pneumothorax before ECMO start, bleeding of 750 mL/day began on day 5, thoracotomy confirmed chest wall drainage channel as source, two additional thoracotomies for massive bleeding related to thoracotomy. Died from thoracic bleeding and circulatory failure | Chest wall (seen at thoracotomy) | 24 Fr standard chest tube | No |
| 5 | Adult | Pneumonia/ARDS | Drainage for clear pleural fluid, bleeding began after 1 day, first externally via the drainage port, then internally with over 1000 mL/day for several days. Conservative management. | Chest wall (outward bleeding) | 8 Fr Seldinger chest tube | No |
| 6 | Pediatric | Pneumonia/ARDS | Drainage for suspected pleural fluid which was more likely a pneumatocele, bleeding of 300 mL/h started shortly after and continued for several hours, large remaining hemothorax treated with new drainage 2 days later. | unknown | 16 Fr standard chest tube | Yes |
| 7 | Adult | Sepsis | Drainage for clear pleural fluid, 3 days later CT showed 15 cm hematoma depressing the diaphragm and contrast extravasation from intercostal artery near drain entry, thoracotomy performed for hematoma evacuation, bleeding continued requiring 12 further thoracotomies including pneumonectomy. | Chest wall (CT contrast extravasation) | 8 Fr Seldinger chest tube | No |
| 8 | Adult | Pneumonia/ARDS | Pleural fluid treated with small drainage, due to malfunction replaced with larger drain over guidewire, perforation of right ventricle caused tamponade requiring emergency sternotomy. | Heart (seen at sternotomy) | 8 Fr Seldinger chest tube | Yes |
| 9 | Adult | Pneumonia/ARDS | Drainage for clear pleural fluid, bleeding started the same day with 900 mL in 24 hours, stopped after heparin pause. | Unknown | 24 Fr Seldinger chest tube | Yes |
| 10 | Neonatal | MAS | Drainage for pneumothorax pre-ECMO, drain accidentally removed on day 5, new CVC placed followed by massive bleeding requiring thoracotomy, bleeding source confirmed in chest wall, possibly initiated by drain removal. | Chest wall  (seen at thoracotomy) | 3 Fr CVC needle | Yes |
| 11 | Neonatal | Sepsis | Bilateral fluid pre-ECMO treated with two PVCs, causing left-sided tension pneumothorax managed with 12 Fr Seldinger drain shortly before ECMO start, massive left-sided bleeding began hours after ECMO initiation, thoracotomy performed through drain channel, no confirmed source but deemed most likely drainage related. | unknown, most likely chest wall | 12 Fr Seldinger chest tube | No |
| 12 | Adult | Pneumonia/ARDS | Drainage for pneumothorax hours before ECMO, bleeding of 1.1 L in 4 hours on ECMO, CT showed chest wall bleeding outward, sutured from outside without thoracotomy. | Chest wall (outward bleeding and CT) | 18 Fr standard chest tube | Yes |
| 13 | Adult | COVID-19 | Two separate bleeding events  1: Drainage for left tension pneumothorax with bleeding and drain clotting, replaced with larger drain via same channel causing massive hemothorax, CT showed likely placement in lung parenchyma.  2: Pig-tail drainage on day 6 for right pleural fluid with dark blood, shortly after output of 2.5 L oxygenated blood, CT showed lung extravasation at drainage level. Bleeding was deemed a major contributor to death. | 1: lung and 2: lung (CT extravasation) | 24 Fr standard chest tube and  6 Fr pigtail | No |
| 14 | Adult | COVID-19 | Drainage for pneumothorax. CT after the procedure showed new large pleural hematoma by the drainage passage. Angiography without extravasation, conservatively treated until drained with pigtail after 18 days | Chest wall (CT) | 20 Fr standard chest tube | Yes |
| 15 | Adult | COVID-19 | Two separate bleeding events  1: Pneumothorax on day 20, Seldinger drain causes liver laceration with massive bleeding - laparotomy.  1: Two new drainages same day in right hemithorax due to remaining pneumothorax. Massive bleeding leading to thoracotomy after 2 days. Bleeding from extra-pleural drainage in chest wall and from drain in lung parenchyma. 2 thoracotomies. Bleeding was deemed a major contributor to death | 1: Liver  2: Chest wall + lung (seen at thoracotomy) | 16 Fr Seldinger chest tube and 24 Fr standard chest tube | No |
| 16 | Adult | Pneumonia/ARDS | Lung abscess treated with pigtail drain. Second day 1.6 L bleeding mainly outwards. | Chest wall (outward bleeding) | 10 Fr pigtail | Yes |
| 17 | Adult | COVID-19 | Drainage for pneumothorax with 700 mL bleeding before drain dislocated, two additional drainages for large hemothorax, CT showed chest wall extravasation treated with coiling, remaining hematoma later managed with drains and intrapleural rTPA. | Chest wall (CT extravasation) | 12 Fr Seldinger chest tube | Yes |
| 18 | Adult | COVID-19 | Drainage placed before ECMO for pneumothorax, accidentally dislocated on day 26, next day required transfusions, new drainage placed after 2 days with total bleeding of 900 mL, bleeding stopped after heparin discontinuation. | Unknown | 16 Fr standard chest tube | No |
| 19 | Pediatric | Sepsis | Drain placed for clear pleural fluid directly after VA ECMO start, on day 4 bleeding from drain incision mainly outward and via drain, 585 mL in one day with transfusion of 5 units PRBC, bleeding stopped after heparin discontinuation. | Chest wall (outward bleeding) | 8 Fr Seldinger chest tube | Yes |
| 20 | Neonatal | Pneumonia/ARDS | Bilateral pleural drainages for clear fluid with cardiac compression and mediastinal shift, left-sided bleeding began on day 6 with multiple transfusion need, CT showed chest wall extravasation at drainage entry, bleeding stopped spontaneously. | Chest wall (CT extravasation) | 8 Fr Seldinger chest tube | No |
| 21 | Adult | Intoxication | Suspected tension pneumothorax on day 3, two Seldinger punctures without air return, 3 hours later circulatory instability with massive hemothorax evacuated by drainage, CT confirmed thoracic wall arterial bleeding treated with coiling and thoracotomy, both with limited effect, 16 L transfused, treatment discontinued, bleeding deemed major contributor to death. | Chest wall (CT extravasation) | 5 Fr Seldinger micro puncture kit | No |
| ARDS: acute respiratory distress syndrome; CVC: central venous catheter; CT: computed tomography; ECMO: extracorporeal membrane oxygenation; Fr: French (catheter size); MAS: meconium aspiration syndrome; PRBC: packed red blood cells; rTPA: recombinant tissue plasminogen activator; SD: standard deviation; VA: veno-arterial | | | | | | |
