# Supplementary material for: Bleeding Complications from Chest Tube Treatment in Patients on Extracorporeal Membrane Oxygenation Support
Source: Interdiscip Cardiovasc Thorac Surg. 2025 Nov 22;40(11):ivaf271. doi: 10.1093/icvts/ivaf271 (PMC12653765; doi:10.1093/icvts/ivaf271)
Supplement: ivaf271_Supplementary_Data [file ivaf271_supplementary_data.zip › Supplementary Table 2 and 3.docx]

| **Supplementary Table 2.** Bivariate logistic regression of patient-level baseline characteristics for major chest-tube bleeding | | | |
| --- | --- | --- | --- |
| Variable | Odds ratio (95% CI) | p-value |  |
|  |  |  |  |
| Age (per 1 year) | 1.02 [1.00–1.04] | 0.025 |  |
| Female sex (vs male) | 0.37 [0.13–1.08] | 0.068 |  |
| Weight (per 1 kg) | 1.01 [1.00–1.02] | 0.043 |  |
| Year group |  |  |  |
| 2010–2014 | ref | - |  |
| 2015–2020 | 0.85 [0.24–2.99] | 0.797 |  |
| 2021–2024 | 0.33 [0.06–1.91] | 0.215 |  |
| Number of chest tubes (per 1 tube) | 2.46 [1.59–3.81] | <0.001 |  |
| CI: confidence interval | | | |

| **Supplementary Table 3.** Bivariate logistic regression of individual chest tubes factors for major chest-tube bleeding | | | |
| --- | --- | --- | --- |
| Variable | Odds ratio (95% CI) | p-value |  |
| Chest tube placed on ECMO (vs pre-ECMO) | 2.58 [0.98–6.77] | 0.054 |  |
| Mode of ECMO |  |  |  |
| Pre-ECMO | ref | - |  |
| VA | 2.34 [0.82–6.68] | 0.113 |  |
| VV | 3.03 [0.97–9.50] | 0.057 |  |
| Laterality right side (vs left) | 0.79 [0.33–1.88] | 0.599 |  |
| Type of drain, n (%) |  |  |  |
| Standard chest tube (open) | ref | - |  |
| Seldinger chest tube (guide wire) | 0.92 [0.31–2.70] | 0.878 |  |
| Pigtail drainage/CVC/PVC | 1.18 [0.43–3.20] | 0.749 |  |
| Indication pneumothorax (vs fluid) | 0.94 [0.39–2.26] | 0.897 |  |
| CI: confidence interval; CVC: central venous catheter; ECMO: extracorporeal membrane oxygenation; PVC: peripheral venous catheter; VA: venoarterial; VV: venovenous | | | |
